# A conserved stem of the *Myxococcus xanthus* sRNA Pxr controls sRNA accumulation and multicellular development

**DOI:** 10.1038/s41598-017-15439-w

**Published:** 2017-11-13

**Authors:** Yuen-Tsu N. Yu, Elizabeth Cooper, Gregory J. Velicer

**Affiliations:** 1 Institute of Integrative Biology, ETH Zurich, Universitätstrasse 16, 8092 Zurich, Switzerland; 2 Department of Biology, Indiana University, Bloomington, IN 47405 USA

## Abstract

The small RNA (sRNA) Pxr negatively controls multicellular fruiting body formation in the bacterium *Myxococcus xanthus*, inhibiting the transition from growth to development when nutrients are abundant. Like many other prokaryotic sRNAs, Pxr is predicted to fold into three stem loops (SL1-SL3). SL1 and SL2 are highly conserved across the myxobacteria, whereas SL3 is much more variable. SL1 is necessary for the regulatory function of Pxr but the importance of SL3 in this regard is unknown. To test for *cis* genetic elements required for Pxr function, we deleted the entire *pxr* gene from a developmentally defective strain that fails to remove Pxr-mediated blockage of development and reintroduced variably truncated fragments of the *pxr* region to test for their ability to block development. These truncations demonstrated that SL3 is necessary for Pxr function in the defective strain. We further show that a highly conserved eight-base-pair segment of SL3 is not only necessary for Pxr to block development in the defective strain under starvation conditions, but is also required for Pxr to prevent fruiting body development by a developmentally proficient wild-type strain under high-nutrient conditions. This conserved segment of SL3 is also necessary for detectable levels of Pxr to accumulate, suggesting that this segment either stabilizes Pxr against premature degradation during vegetative growth or positively regulates its transcription.

## Introduction

Small RNAs (sRNA) play major roles in bacterial gene regulation^1–3^, including in the Gram-negative myxobacteria, which cooperatively construct multicellular fruiting bodies in response to starvation^4,5^. By base-pairing with mRNAs, sRNAs can modulate protein translation and mRNA degradation. The sRNA Pxr in the model myxobacterium *Myxococcus xanthus* was previously found to regulate the transition from vegetative growth when nutrients are abundant to the initiation of fruiting body development upon starvation^4^. Deletion of *pxr* from the developmentally proficient lab reference strain GJV1 causes robust fruiting body development and high levels of spore production when growth substrate is abundant, whereas GJV1 undergoes development only when growth resources are scarce or absent. Pxr is produced in two forms, one long (Pxr-L) and one short (Pxr-S), with Pxr-L presumably being processed by a ribonuclease to Pxr-S. Pxr-S appears to be the active form of Pxr blocking development, as Pxr-S levels are rapidly diminished in response to early developmental signals upon onset of starvation whereas Pxr-L remains present at high levels throughout development^4^.

The *pxr* gene appears to have originated in the base lineage of the Myxococcales suborder Cystobacterineae, as *pxr* homologs were found in almost all examined species within this suborder^6^. Divergent homologs from several Cystobacterineae species, including *Stigmatella aurantiaca*, as well as the inferred ancestral allele, have been shown to effectively control development in *M*. *xanthus* ^7^, thus indicating that *pxr* is likely to play a similar role in controlling development across species. *pxr* appears to have co-evolved with genes encoding a two-component regulatory system that are present upstream of *pxr* in all myxobacterial species examined to date^8^. This conserved two-component system (*pxrR*/*pxrK*) has been shown to regulate Pxr synthesis and processing^8^.

Pxr was identified due to a spontaneous mutation that restored developmental proficiency to the developmentally defective strain OC^9^. OC is a descendant of the developmentally-proficient lab reference strain GJV1 and evolved during 1000 generations of vegetative growth in a high-nutrient liquid environment^10^. During that evolution experiment, the OC lineage accumulated 14 mutations^11^ and concomitantly lost the ability to effectively develop and produce heat-resistant spores^10^. During a subsequent competition experiment, a spontaneous mutation in an OC cell restored developmental proficiency to the resulting mutant, PX^9^. That mutation occurred within the loop of the first of three predicted stem-loop structures (SL1-SL3) of Pxr and inactivated Pxr function^4^.

**Figure 1:**
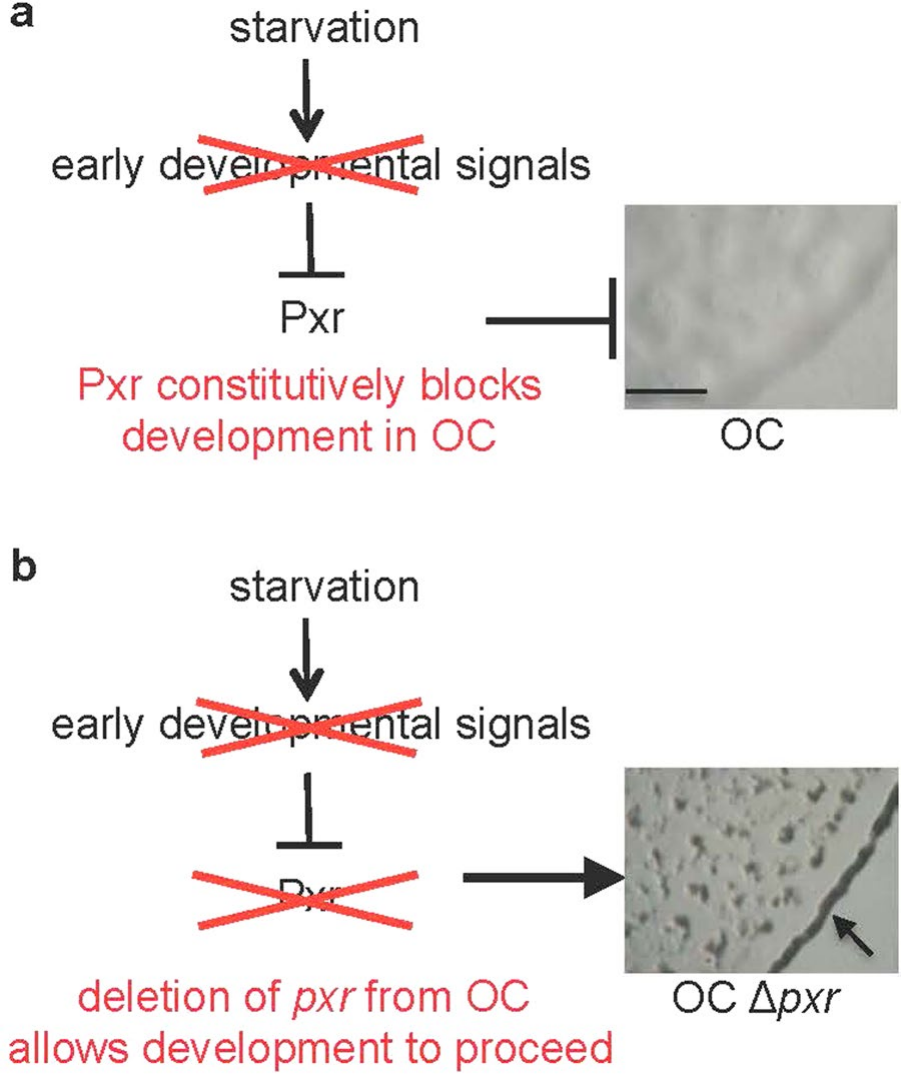
Model of the Pxr-mediated defect in OC development. (**a**) In this model, strain OC is developmentally defective (no fruiting body formation on starvation plates, see image on right (scale bar ~1 mm)) because it fails to relay an early developmental signal that normally deactivates the functional form of Pxr sRNA, which blocks development during vegetative growth in developmentally proficient strains. Therefore, Pxr remains present in OC even under starvation and thereby continues to block development. (**b**) Deletion of the Pxr coding gene from OC alleviates negative regulation and thus allows development to proceed. In the image to the right, fruiting bodies form on starvation plates (especially clusters of intertwined fruiting bodies linked together at the edge of the spotted starving population that forms a darkened circle indicated by an arrow).

Strain OC is defective at development because Pxr-mediated blockage of development is not alleviated upon starvation (Fig. 1a). Deletion of *pxr* from OC removes that blockage and allows development to proceed^4^ (Fig. 1b), as does either deletion of the SL1 loop^8^ or a single-base substitution in the fifth base of that loop (C to A)^4^. Thus, Pxr SL1 is essential for control of *M*. *xanthus* development. While the functional roles of specific sequence elements in some non-coding regulatory sRNAs in other bacteria have been explored extensively^12^, it remains unknown whether other segments of the predicted Pxr structure other than SL1 are necessary for Pxr function. Here we examine the functional effects of a series of deletions from the 3′ end of the *pxr* coding region, including deletions that remove part or all of SL3, as well as deletion of a highly conserved eight base-pair segment of the SL3 stem.

## Results

### The third stem loop of Pxr is required for control of development

**Figure 2:**
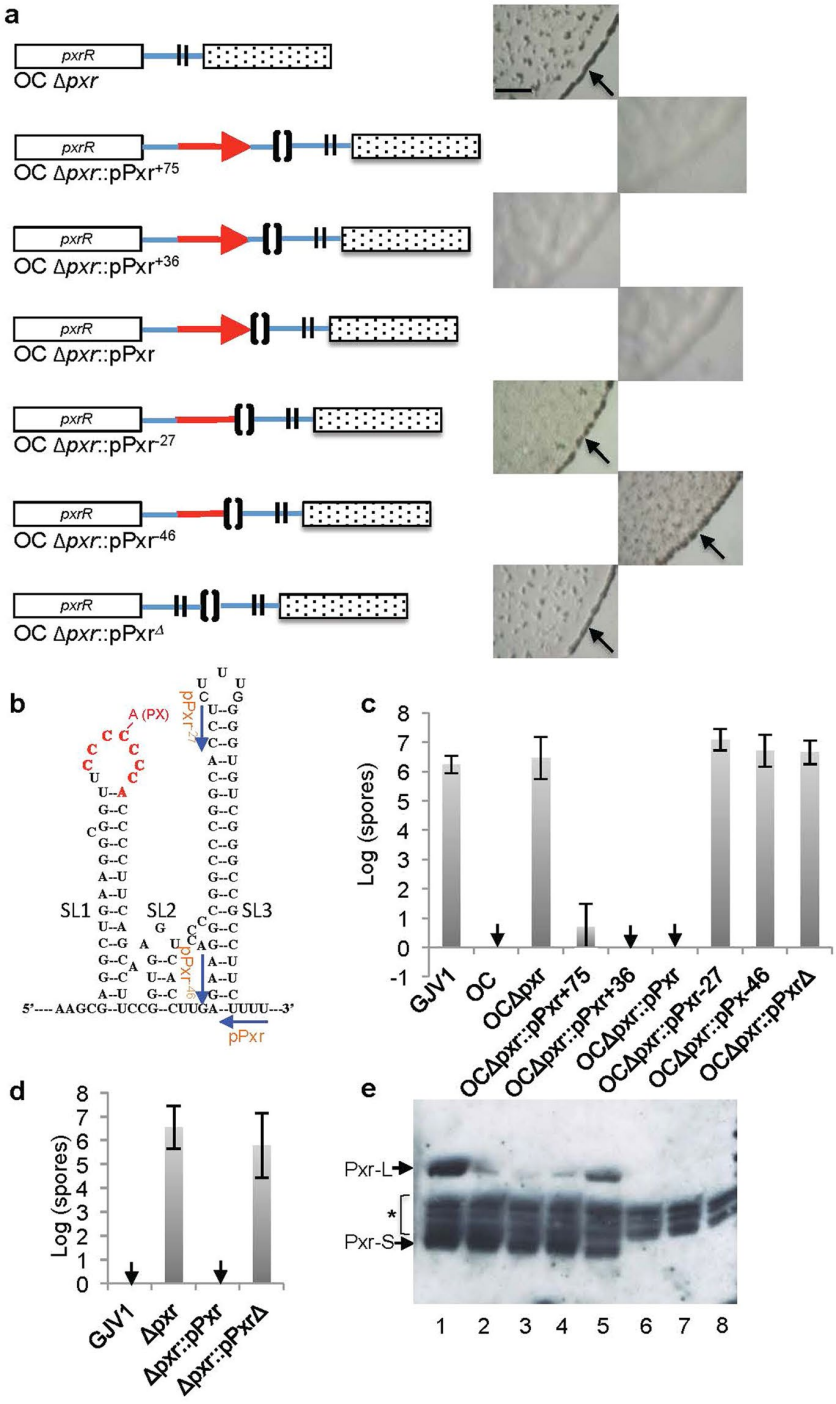
Pxr SL3 is necessary for inhibition of development and accumulation of Pxr. (**a**) A diagram of the *pxr* region for each *pxr* derivative (left) and each strain’s corresponding developmental phenotype on TPM plates (right). The opened and dotted rectangles represent the *pxrR* and *Mxan_1079* genes, respectively. Red arrows represent the annotated *pxr* coding region and red lines without an arrow indicate truncated alleles. Blue lines indicate intergenic regions. The positions from which *pxr* was deleted are represented by double vertical lines and the positions of plasmid integration are represented by brackets. Strains lacking *pxr* (OC Δ*pxr*, OC Δ*pxr*::pPxr^Δ^) or part or all of SL3 (OC Δ*pxr*::pPxr^−27^, OC Δ*pxr*::pPxr^−46^) produce darkened fruiting bodies (indicated by arrows) whereas the three strains containing complete copies of *pxr* (OC Δ*pxr*::pPxr, OC Δ*pxr*::pPxr^+36^, OC Δ*pxr*::pPxr^+75^) do not (scale bar ~1 mm). (**b**) The predicted secondary structure of Pxr. The locations of the C-A substitution and 8-nt deletion that restore high sporulation to strain OC^4,9^ are shown in red. The positions of 3′ primers used for generating respective *pxr*-truncated constructs (pPxr, pPxr^−27^ and pPxr^−46^) are shown with blue arrows. (**c**) Deletion of *pxr* from OC allows OC Δ*pxr* to restore sporulation, whereas integration of three plasmids carrying the entire *pxr* gene (pPxr, pPxr^+36^ and pPxr^+75^) suppresses sporulation. Integration of plasmids carrying *pxr* fragments lacking either the entire third stem-loop sequence (pPxr^−46^) or only the third loop and right side of the corresponding stem (pPxr^−27^) fails to control development, as does integration of the vector-only control pPxr^Δ^. Error bars represent 95% confidence intervals and black downward arrows indicate the absence of spores at the limit of detection (also in Figs 2d and 4a). (**d**) GJV1 Δ*pxr* can sporulate in the presence of abundant nutrients while GJV1 cannot. The introduction of a functional copy of *pxr* restores Pxr-mediated blockage of development at high nutrient levels. (**e**) Pxr accumulation patterns from Northern-blot analysis of strains GJV1 (lane 1), OC Δ*pxr*::pPxr^+36^ (lanes 2 and 3), OC Δ*pxr*::pPxr^+75^ (lane 4), OC Δ*pxr*::pPxr (lane 5), OC Δ*pxr*::pPxr^−27^ (lane 6), OC *pxr*::pPxr^−46^ (lane 7) and OC Δ*pxr* (lane 8). The asterisk indicates bands due to non-specific binding to the Pxr probe.

We constructed several plasmids carrying sequences that all begin at the same position within the 3′-terminal region of the *pxrR* gene (previously known as *Mxan_1078* and *nla19*)^8^. *pxrR* lies immediately upstream of *pxr* and is predicted to encode an NtrC-like response regulator that is part of a two-component signal transduction system along with the predicted histidine kinase gene *pxrK* (previously known as *Mxan_1077*)^8^. These plasmids extend variable lengths to include either the entire predicted *pxr* coding region (pPxr, pPxr^+36^ and pPxr^+75^) or only 5′ portions of *pxr* lacking part or all of SL3 (pPxr^-27^ and pPxr^−46^). Plasmids pPxr^+36^ and pPxr^+75^ include sequence extending 36 and 75 bases beyond the predicted 3′ end of *pxr*, respectively. Additionally, a plasmid carrying sequence also starting from the same position in *pxrR* as the other plasmids but not including the *pxr* coding sequence (pPxr^Δ^) was constructed as a control to test for any effect of plasmid-vector integration. All of these plasmids were integrated into a mutant of strain OC from which the *pxr* coding sequence was deleted (OC Δ*pxr*) but which still retains the intergenic sequence between *pxrR* and *pxr*, thus creating merodiploids for the *pxr* region (Fig. 2a). All of the resulting transformants share the same 167 bp 3′ terminal sequence of *pxrR* and the 260-bp region upstream of *pxr*, including its predicted σ^54^ promoter and the upstream enhancer binding region.

As reported previously^4^, deletion of *pxr* from OC (strain OC Δ*pxr*) restores fruiting body development and spore production to high levels relative to OC (which fails to aggregate into visible fruiting bodies and makes zero or few spores under standard developmental conditions (Figs 1 and 2a,c)). As expected, integration of the negative control plasmid pPxr^Δ^ lacking *pxr* into OC Δ*pxr* did not eliminate fruiting body formation or reduce spore production in the resulting transformant (Fig. 2a,c). Additionally, integration of all three plasmids carrying the entire *pxr* coding region (pPxr, pPxr^+36^ and pPxr^+75^) into OC Δ*pxr* resulted in blockage of both fruiting body formation (Fig. 2a) and spore production (Fig. 2c), indicating that *pxr* is functionally expressed. Unlike complete copies of *pxr*, fragments of *pxr* lacking half or all of SL3 (pPxr^−27^ and pPxr^−46^, respectively) were unable to block development (Fig. 2a). Transformants produced spores at levels similar to those of GJV1 and OC Δ*pxr* (Fig. 2c).

The experiments above were all performed in the context of the OC genomic background, which differs from its evolutionary ancestor GJV1 by 14 known mutations^11^. As shown both previously^4^ and here, deletion of *pxr* from GJV1 (creating strain GJV1 Δ*pxr*) causes development to proceed and produces heat-resistant spores in the presence of abundant nutrients (e.g. 0.3% casitone, Fig. 2d), indicating that *pxr* controls the transition of *M*. *xanthus* from growth on abundant nutrients to development in response to starvation. To demonstrate that the results above are not likely to be specific to the OC background, we introduced a full copy of *pxr* on plasmid pPxr into strain GJV1 Δ*pxr*. If the introduced *pxr* is active and performs the same function as the original native *pxr*, fruiting body development and spore production should be blocked. Indeed this is the case, as integration of pPxr restored *pxr* blockage of high-nutrient development, whereas integration of the corresponding plasmid vector lacking any *pxr* sequence (pPxr^Δ^) did not (Fig. 2d).

### The third stem loop of Pxr is essential for Pxr accumulation

Consistent with our developmental assays, integration of all three plasmids carrying the entire *pxr* gene into OC Δ*pxr* resulted in accumulation of high levels of Pxr-S, the short active form of Pxr that negatively regulates development (Fig. 2e, lanes 2–5). In contrast, integration of the two plasmids lacking part or all of SL3 (pPxr^−27^ and pPxr^−46^), yielded no visible Pxr transcript of the expected sizes (Fig. 2e, lanes 6–7). Rather, the respective transformants showed the same transcriptional profile as the control strain lacking *pxr* (Fig. 2e, lane 8), thus suggesting that the Pxr transcripts lacking part or all of SL3 may be unstable.

Interestingly, levels of the long form of Pxr, Pxr-L, were much lower in the merodiploids transformed with plasmids that included both the entire predicted *pxr* gene and a downstream extension (Fig. 2e, lanes 2–4) than were present in the transformant carrying only *pxr* with no extension (Fig. 2e, lane 5). This result implies that the region extending 36 bp downstream of the predicted *pxr* coding sequence reduces Pxr-L accumulation, but only in the context of these merodiploid transformants of OC Δ*pxr*. Additionally, the accumulated levels of Pxr-L in all three merodiploids (Fig. 2e, lanes 2–5) bearing the *pxr* gene were significantly lower than that found in the control strain GJV1 (Fig. 2e, lane 1). This reduction may be due to the second copy of the *pxr* promoter and the upstream enhancer-binding region preceding the *pxr*-deleted allele in the merodiploids (Fig. 2a) that may sequester important transcription factors such as PxrR and sigma factor away from transcribing the functional Pxr.

### A conserved 8-bp segment of Pxr SL3 is essential for blockage of development in both the OC and GJV1 genomic backgrounds

**Figure 3:**
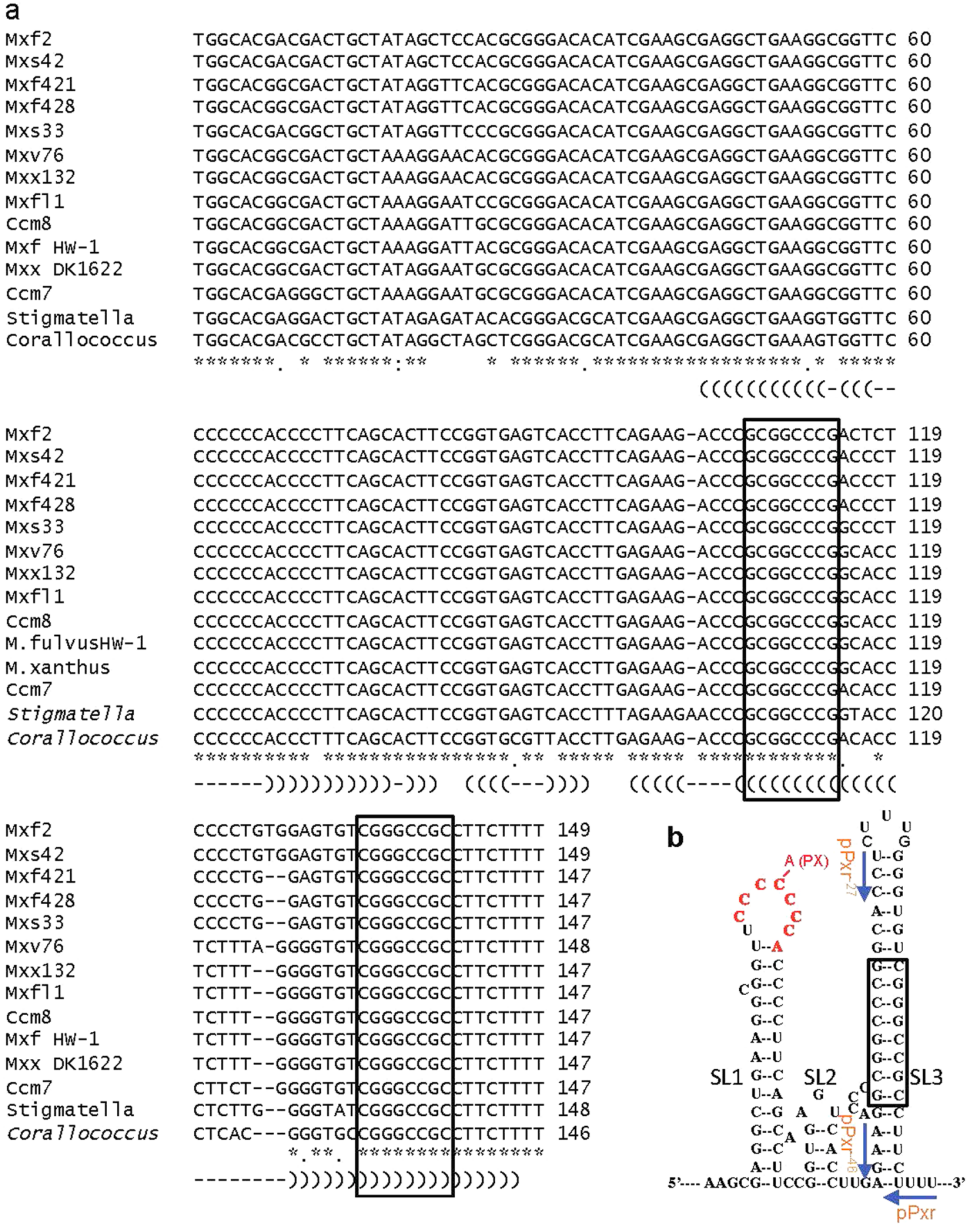
Comparative analysis of *pxr* sequences across the myxobacteria reveals a highly conserved segment of the predicted third stem-loop structure. (**a**) Sequence alignment of 14 myxobacterial *pxr* homologs. Two segments of eight bases (boxed regions) that are complementary are predicted to form the core of the third Pxr stem and are conserved across nine highly diverse species of myxobacteria, whereas other portions of this stem and the corresponding loop are less conserved. (*Corallococcus* = *Corallococcus collaroides* strain DSM 2259, Ccm = *M*. *macrosporus*, Mxf = *M*. *fulvus*, Mxfl = *M*. *flavescens*, Mxs = *M*. *stipitatus*, Mxv = *M*. *virescens*, *Stigmatella = Stigmatella aurantiaca* strain DW4/3–1. Strain sources are listed in Table 1 of Chen *et al*.^6^. Asterisks indicate bases conserved across all homologs. Parentheses indicate complementary pairing. (**b**) The diagram shows the position of the highly conserved 8-bp stem (boxed) located in the third stem loop of the predicted Pxr secondary structure.

**Figure 4:**
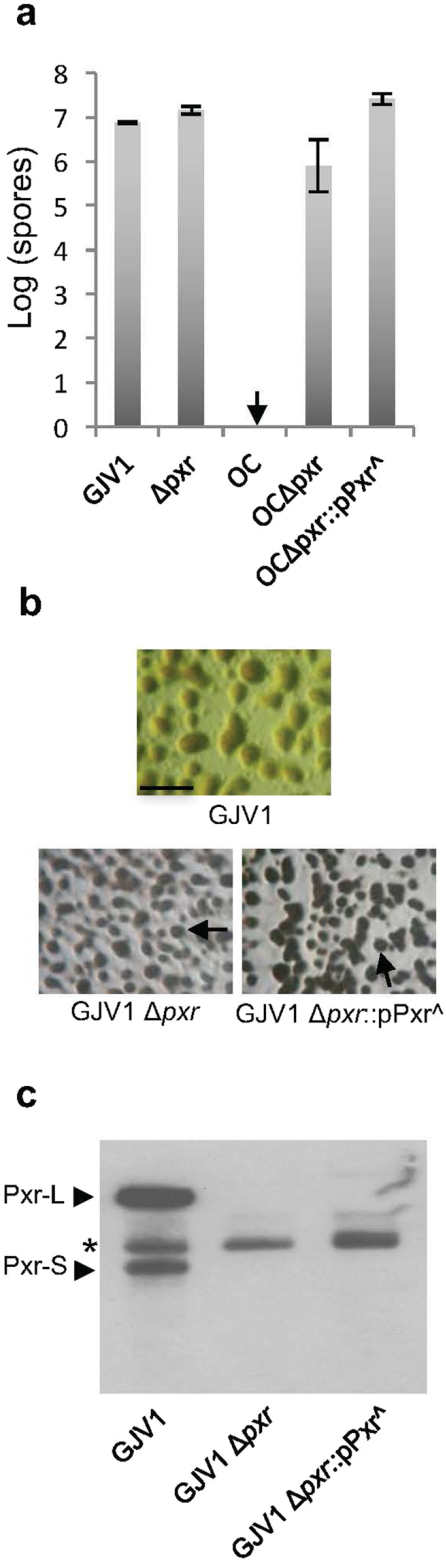
The conserved 8-bp portion of the Pxr SL3 stem (SL3:8 bp) is necessary for Pxr function. (**a**) An allele of *pxr* lacking the conserved 8-bp segment (*pxr^*) fails to block sporulation when integrated into OC Δ*pxr*. (**b**) Developmental phenotypes on 0.3% casitone CTT agar. GJV1 Δ*pxr* and GJV1 Δ*pxr*::pPxr^ form darkened fruiting bodies (a single fruiting body is indicated by an arrow) whereas GJV1 does not. GJV1 forms only translucent mounds, but not opaque fruiting bodies (scale bar ~1 mm). (**c**) Northern blot analysis of Pxr accumulation in liquid CTT medium. Pxr transcript does not accumulate to visibly detectable levels in GJV1 Δ*pxr*::pPxr^ but is present at high levels in both forms (Pxr-L and Pxr-S) in GJV1. The asterisk indicates bands due to non-specific binding to the Pxr probe.

Comparison of *pxr* sequences across a phylogenetically diverse range of myxobacterial species revealed a highly conserved 8-bp segment within the Pxr SL3 stem (Fig. 3, the boxed region in the sequence alignment and within the simulated SL3). We therefore tested whether this stem segment is necessary for Pxr function by constructing an allele of *pxr* lacking the respective 16 bases (*pxr*^, Fig. 3b). Integrating of the plasmid carrying this allele (pPxr^) into OC Δ*pxr* failed to block sporulation (Fig. 4a). Thus, like the two *pxr* alleles lacking either the entire SL3 sequence (*pxr* ^*−*46^) or the 3′-terminal half of SL3 (*pxr* ^*−*27^) (Fig. 2a,c), *pxr*^ is non-functional and the conserved 8-bp segment of the SL3 stem appears to be essential for Pxr function.

We also asked whether deletion of the conserved segment of SL3 would eliminate Pxr function in the GJV1 genomic background. To do so, we integrated pPxr^ into GJV1 Δ*pxr* and tested for fruiting-body formation under high-nutrient conditions. In the presence of abundant nutrients, GJV1 does not form darkened fruiting bodies due to blockage of development by *pxr* but GJV1 Δ*pxr* does (Fig. 4b). Integration of pPxr^ into GJV1 Δ*pxr* failed to restore the blockage of fruiting body development under high-nutrient conditions and showed the same phenotype as GJV1 Δ*pxr* (Fig. 4b).

Northern-blot analysis revealed that the 8-bp conserved segment of SL3 is necessary for accumulation of Pxr transcript, as no Pxr transcript of any size was observed for GJV1 Δ*pxr*::pPxr^ (Fig. 4c). This segment of the SL3 stem might affect Pxr-transcript accumulation either by stabilizing Pxr-L once it has been produced or by facilitating transcription of *pxr*.

## Discussion

Non-coding sRNAs primarily modulate expression of target genes by base-pairing to their mRNAs and thus causing mRNA degradation or altered protein translation^1,2^. Pxr sRNA is highly expressed in the vegetative stage and processed into the putative active smaller form (Pxr-S) to presumably either directly repress developmental genes or enhance the expression of other developmental inhibitors when nutrients are abundant^4^. However, the direct targets of Pxr (if multiple) and their relationships to genes known to positively regulate the early stages of development remain under investigation.

To facilitate better understanding of how Pxr regulates development, we sought to identify segments pivotal to its function. Our results demonstrate the necessity of Pxr SL3 for regulating the Pxr-mediated developmental pathway. Most studies examining functional components of sRNA regulators have focused on sRNA interactions with regulatory targets (e.g. sequences involved in sRNA binding)^13–15^. In the case of Pxr, however, we have identified a highly conserved 8-bp segment within the third predicted stem loop (SL3:8 bp) that appears to be essential in maintaining levels of Pxr sufficient for effective negative regulation of fruiting body development. In particular, deletion of the invariable 8-bp stem abolishes the accumulation of Pxr transcripts and results in a *pxr*-null phenotype.

Comparison across all known *pxr* alleles exclusively identified in myxobacteria showed that the most variable sequences are located in the annotated SL3 (despite the highly conserved 8-bp segment core) while SL1 and SL2 are much more conserved as a whole^6^. Mutation or deletion within the loop of SL1 eliminates the negative regulatory function of *pxr*, but does not prevent accumulation of high levels of Pxr transcript, indicating that SL1 plays a key role in Pxr functionality, but not in its transcription or in its stability^4,8^. Such SL1-mediated Pxr functionality may involve target pairing or RNA-chaperone binding (although *M*. *xanthus* does not carry a homolog of *hfq* ^16^).

In contrast to the effects of a deletion in the SL1 loop^8^, deletion of SL3:8 bp in the GJV1 background strongly affects sRNA accumulation (Fig. 4c), which reflects a balance between sRNA synthesis and decay. The necessity of SL3:8 bp for Pxr accumulation might be due to an autoregulatory effect on transcription, as occurs with some other sRNAs^17,18^. However, there is no evidence to suggest this scenario. In particular, the response regulator *pxrR*, which has been shown to regulate *pxr* transcription^8^, is not predicted to be a target of Pxr (unpublished results).

Alternatively, it is possible that SL3:8 bp is a critical determinant of Pxr stability, such that Pxr transcripts of *pxr^* are synthesized but rapidly degrade and are thus not detected on Northern blots. If the SL3:8 bp segment is crucial in protecting the transcripts from ribonuclease attack either by shielding the RNA from ribonuclease binding or by interacting with protective proteins, it is expected that deletion of SL3:8 bp will severely diminish Pxr accumulation. In *E*. *coli*, Hfq together with ribonucleases such as PNPase (polynucleotide phosphorylase) and RNasePH have been shown to stabilize some sRNAs and facilitate regulation of their targets^19,20^. In *Pseudomonas putita*, Hfq and Crc bind to CrcZ sRNA and protect it from degradation^21^. However, in those studies the sRNA regions conferring protection against degradation remain elusive. It has been documented that Hfq can recognize the 3′-polyU region of SgrS sRNA to maintain its stability^22^. While there is no homolog of *hfq* gene found in myxobacteria, it is possible that some myxobacteria proteins other than Hfq can perform a chaperone-like function to promote sRNA stability. Further analysis of the functional role of SL3:8 bp may identify any such chaperone-like proteins and provide further insights regarding how levels of the myxobacterial sRNA Pxr are regulated during the transition from growth to development.

## Methods

Strain and plasmid descriptions and primer sequences used in this work are listed in Table 1.

**Table 1:** Plasmid, strain and primer information.

Plasmid	Description	Reference
pCR-Blunt	Cloning/integrative vector	Invitrogen
pPxr^+75^	The insert is amplified by primers GV367 + 371. It contains 167 bp of *Mxan_1078* 3′-terminus, the entire 413 bp intergenic region bearing the entire *pxr* coding sequence and 32 bp of the *Mxan*_1079 5′- terminus.	This work
pPxr^+36^	The insert is amplified by primers GV367 + 492. It contains 167 bp *of Mxan_1078* 3′-terminus and 407 bp of intergenic sequence ending 7 bp upstream of the *Mxan_1079* start codon. It bears the entire *pxr* coding sequence.	This work
pPxr	The insert is amplified by primers GV367 + 611. It contains 167 bp of *Mxan_1078* 3′-terminus and 370 bp of intergenic sequence ending 44 bp upstream of the *Mxan_1079* start codon. It bears the entire predicted *pxr* coding sequence.	This work
pPxr^−27^	The insert is amplified by primers GV367 + 612. It contains 167 bp of *Mxan_1078* 3′-terminus and 343 bp intergenic sequence ending 71 bp upstream of the *Mxan_1079* start codon. It is missing the second half of SL3.	This Work
pPxr^−46^	The insert is amplified by primers GV367 + 613. It contains 167 bp of *Mxan_1078* 3′-terminus and 324 bp intergenic sequence ending 90 bp upstream of the *Mxan_1079* start codon. It is missing most of SL3.	This work
pPxr^Δ^	The insert is amplified by primers GV367 + 490. It contains 167 bp of *Mxan_1078* 3′-terminus and 260 bp of intergenic sequence ending 154 bp upstream of the *Mxan_1079* start codon. It is missing the entire *pxr* coding sequence.	This work
pPxr^	The insert is amplified by primers GV367 + 683. It contains 167 bp of the *Mxan_1078* 3′-terminus and 354 bp of intergenic sequence ending 44 bp upstream of the *Mxan_1079* start codon. Is it missing the 8-bp conserved segment of the SL3 stem (SL3:8 bp).	This work
**Strain**	**Description**	**Reference**
Top10	*E*.*coli* strain for plasmid construction	Invitrogen
GJV1	WT *M*. *xanthus* strain, a derivative of DK1622	10,18
GJV1 Δ*pxr*	GJV1 with the entire *pxr* gene deleted	4
OC	Kanamycin-resistance marked derivative of obligate cheater strain GVB207.3 (aka GJV32)	4,9
GVB207.3	unmarked version of OC	4,9
OC Δ*pxr*	GVB207.3 with the entire *pxr* gene deleted	4
OC Δ*pxr*::pPxr^+75^	OC Δ*pxr* with plasmid pPxr^+75^ integrated at the native *pxr* locus	This work
OC Δ*pxr*::pPxr^+36^	OC Δ*pxr* with plasmid pPxr^+36^ integrated at the native *pxr* locus	This work
OC Δ*pxr*::pPxr^Δ^	OC Δ*pxr* with plasmid pPxr^Δ^ integrated at the native *pxr* locus	This work
OC Δ*pxr*::pPxr	OC Δ*pxr* with plasmid pPxr integrated at the native *pxr* locus	This work
OC Δ*pxr*::pPxr^−27^	OC Δ*pxr* with plasmid pPxr^−27^ integrated at the native *pxr* locus	This work
OC Δ*pxr*::pPxr^−46^	OC Δ*pxr* with plasmid pPxr^−46^ integrated at the native *pxr* locus	This work
OC Δ*pxr*::pPxr^	OC Δ*pxr* with plasmid pPxr^ integrated at the native *pxr* locus	This work
GJV1 ::pPxr	GJV1 with plasmid pPxr integrated at the native *pxr* locus	This work
GJV1 ::pPxr^Δ^	GJV1 with plasmid pPxr^Δ^ integrated at the native *pxr* locus	This work
GJV1 Δ*pxr*::pPxr	GJV1 Δ*pxr* with plasmid pPxr integrated at the native *pxr* locus	This work
GJV1 Δ*pxr*::pPxr^Δ^	GJV1 Δ*pxr* with plasmid pPxr^Δ^ integrated at the native *pxr* locus	This work
GJV1 Δ*pxr*::pPxr^	GJV1 Δ*pxr* with plasmid pPxr^ integrated at the native *pxr* locus	This work
**Primer**	**Sequence**	
GV367	CCCAGGTGGTGGAAGAGG	
GV371	CGCAGCACCCACTGAGATTC	
GV492	CTTTCGTCGCGAGCCGAG	
GV490	CGATGTGTCCCGCGCATTCC	
GV611	AAAAGAAGGCGGCCCGACAC	
GV612	AGGTGCCGGGCCGCGGGTC	
GV613	TTCTCAAGGTGACTCACCGG	
GV683	AAAAGAAGACACCCCAAAGAGGTGCG	
	GGTCTTCTCAAGGTGACTCACC	

### Development assays and sporulation efficiency

The developmental phenotypes of the *pxr*-derivative strains as well as the controls were examined on TPM^23^ 1.5% agar starvation plates. Mid-log phase cultures grown in CTT^24^ medium were centrifuged and resuspended in TPM liquid to a density of ~5 × 10^9^ cells/ml. 50 µl of resuspended culture were spotted onto TPM agar plates and incubated at 32 °C. After four or five days, images of developmental phenotypes were taken with a Zeiss Stemi 2000 stereomicroscope. At day five of development the developing populations were harvested into 1 ml of ddH_2_O, incubated at 50 °C for two hours, sonicated twice for 10 seconds to disperse heat-resistant spores. All sonicated samples were serially diluted in ddH_2_O and then plated into CTT soft (0.5%) agar. The number of spores produced by each strain was calculated by multiplying the colony count of germinated spores on CTT soft agar by the relevant dilution factor. Development assays were performed in four temporally independent replicates for the experiments reported in Fig. 2c and two replicates for the experiments reported in Figs 2d and 4a.

### Northern-blot analysis of Pxr sRNA

Pxr sRNA generated from each strain was analysed in a Northern-blot assay described previously^4^. Total RNA was prepared from mid-log phase cultures growing in CTT medium and small RNA fragments (<200 nt) were prepared with the MirVana miRNA isolation kit (Ambion). RNA concentrations were estimated by a NanoDrop spectrophotometer (Thermo Scientific). Equal amounts of RNA (0.7 μg) were electrophoresed in a 10% SequaGel (National Diagnostics). RNA transcripts were electro-transferred onto a BrightStar^®^-Plus positively charged nylon membrane (Ambion) at 180 mA for one hour and were fixed onto the membrane with a UV cross-linker. The membrane was hybridized with 100 pmol 3′Biotin-TEG-*pxr* oligo probe (Sigma, the probe is complementarily base-paired to the sequences within SL1) in 5 ml of UltraHyb-Oligo buffer (Ambion) overnight at 37 °C after completing pre-hybridization in 4 ml of UltraHyb-Oligo buffer for one hour at 37 °C. The positions and relative content of Pxr RNAs were detected and visualized with BrightStar^®^ Biodetect non-isotopic kit (Ambion).

### Plasmid and strain construction

All plasmids were cloned by a similar procedure. The Pxr sRNA coding region is mapped within a 413-bp intergenic region between *Mxan_1078* and *Mxan_1079* ^4,9^. To make plasmids containing a series of the *pxr* 3′-terminal truncations, *pxr* fragments were first PCR-amplified using the same upstream primer located 167-bp upstream of the *Mxan_1078* stop codon (GV367) and a series of downstream primers that generated fragments of desired length (Table 1). The resulting PCR fragments were gel-purified, cloned into the pCR-Blunt (Invitrogen) vector and were verified by sequencing. The downstream primers were designed to generate two fragments containing the complete predicted *pxr* coding region plus extensions of different length beyond the predicted end of *pxr* (pPxr^+75^ and pPxr^+36^), one fragment containing the entire *pxr* gene with no extension (pPxr), two fragments lacking part of the *pxr* gene (pPxr^−27^, which lacks the SL3 loop and the downstream portion of the SL3 stem, and pPxr^−46^, which lacks the entire predicted SL3 sequence) and one fragment lacking the complete *pxr* gene (pPxr^Δ^). To generate pPxr^ bearing the *pxr* derivative devoid of an 8-bp segment of the SL3 stem, a 48-mer primer missing the 16 respective nucleotides (GV683) was used together with GV367 to generate the PCR insert. All of the plasmids described above thus contain a region homologous to the *M*. *xanthus* GJV1/OC genomes (ranging from 427 bp to 612 bp in length) that allows integration via single crossing-over to generate cognate mero-diploid *pxr*-derivative constructs (Fig. 2a). The end positions of the 3′ primers used to generate inserts for plasmids pPxr, pPxr^−27^ and pPxr^−46^ on the predicted three-stem-loop structure of Pxr are shown in Fig. 2b.

## Electronic supplementary material

Supplementary Information
