# Supplementary material for: A conserved stem of the Myxococcus xanthus sRNA Pxr controls sRNA accumulation and multicellular development
Source: Sci Rep. 2017 Nov 13;7:15411. doi: 10.1038/s41598-017-15439-w (PMC5684412; doi:10.1038/s41598-017-15439-w)

Supplementary data

A conserved stem of the *Myxococcus xanthus* sRNA Pxr controls sRNA accumulation and multicellular development

Yuen-Tsu N. Yu[1,2*], Elizabeth Cooper[2,] and Gregory J. Velicer[1, 2*]

[1]*Institute for Integrative Biology, ETH Zurich, Universitätstrasse 16, 8092 Zurich, Switzerland.*

[2]*Department of Biology, Indiana University, Bloomington, IN, USA 47405*

**Correspondence:* nicco.yu@env.ethz.ch or gregory.velicer@env.ethz.ch

Figure legend

Figure S1. The full images of Northern blot assays. a) The full Northern blot image of Fig. 2e. Lanes 1-8 are the same as in Figure 2e. b) The full Northern blot image of Fig. 4c. Lanes 6-8 correspond to the blot shown in Fig. 4c: GJV1 (lane 6), GJV1 Δ*pxr* (lane 7) and GJV1 Δ*pxr*::pPxr^ (lane 8).

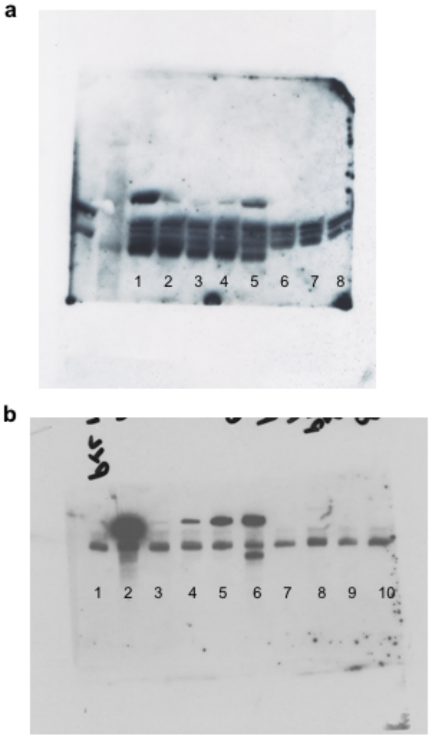

Supplement: Supplementary file 1 — Supplementary Information [file 41598_2017_15439_MOESM1_ESM.pdf]
